# A cross-cultural comparison study of depression assessments conducted in Japan

**DOI:** 10.1186/1744-859X-12-9

**Published:** 2013-04-03

**Authors:** Steven D Targum, Atsuo Nakagawa, Yuji Sato

**Affiliations:** 1 Clintara LLC, 505 Tremont Street, #907, Boston, MA 02116, USA; 2 Massachusetts General Hospital, Boston, MA 02114, USA; 3 Translational Medical Center, National Center of Neurology and Psychiatry and the Department of Neuropsychiatry, Keio University School of Medicine, Tokyo 160-8582, Japan; 4 Center for Clinical Research, Keio University School of Medicine, Tokyo 160-8582, Japan

**Keywords:** Cross-cultural comparison, Depression ratings, Asian culture, MADRS

## Abstract

**Background:**

The advent of global clinical trials has necessitated the use of English-based rating instruments in diverse cultures where English is clearly not the primary language. The cross-cultural applicability of rating instruments developed in one language with only one cultural group is an important issue in both research and clinical settings where these instruments might be used. We examined the cross-cultural applicability of the Montgomery-Asberg Depression Rating Scale (MADRS) in Japan.

**Methods:**

As part of a rater-training program for a clinical trial in Japan, we assessed inter-rater agreement using two videotaped MADRS interviews administered in Japanese and produced with English subtitles. We looked for possible interpretational variance that might have been generated by cultural differences between Japanese raters in Japan and English-speaking raters in the USA scoring the same interviews.

**Results:**

The US and Japanese raters demonstrated high inter-rater agreement and no significant scoring difference on the total MADRS score. The subtitles in English did not adversely affect the overall scoring.

We separately analyzed the 10 individual items from each of the two MADRS interviews used for rater training. Of the 20 items, 18 were concordant between the US and Japanese raters. In one interview, the US raters scored lassitude significantly higher (*p* = 0.013) and the inability to feel significantly lower (*p* = 0.037) than the Japanese raters, reflecting a possible interpretational difference on these items.

**Conclusion:**

Although developed in Europe, these findings support the general applicability of the MADRS to assess the severity of depressive symptoms in Japan. We did note significant scoring differences on 2 of the 20 individual items, suggesting a possible cultural difference. It is possible that more interviews might have revealed more interpretational differences. These findings highlight the need for cultural familiarity when assessing psychiatric patients.

## Background

Central nervous system clinical trials rely on reliable and valid rating instruments to evaluate the efficacy of candidate drugs. These instruments have been translated and validated in many languages in order to facilitate the local (native) languages that are required in multi-national trials. Generally, the training materials and demonstration interviews used to establish inter-rater reliability for rater certification are developed in English with subtitles provided in the requisite local languages. It has been presumed that demonstration interviews using instruments developed in Western countries (Europe or the USA), and conducted with English-speaking subjects from Western countries, can be used to effectively train and standardize scoring among raters from non-Western nations. Clearly, there are cultural differences in the assessment of psychiatric patients that can influence ratings and adversely affect inter-rater agreement [1-4]. Mackin et al. [4] showed significant cultural differences in the assessment of acutely manic subjects when the same videotaped interviews were shown to English-speaking raters from India, the UK, and the USA. They emphasized that effective training materials need to be culturally ‘neutral’ in order to achieve inter-rater agreement. However, there have been few studies that examined the inter-rater agreement of subtitled interviews across cultures that did not speak English as a primary language. Kalali et al. [3] noted cultural differences on the subtitled Positive and Negative Syndrome Scale (PANSS) interviews during rater training programs conducted across six Eastern European countries speaking six different languages. Alternatively, other studies [5-7] have reported high concordance rates using subtitled PANSS and the NSA-16 (Negative Symptom Assessment scale) interviews with non-English-speaking raters from multiple countries including Eastern Europe and Southeast Asia. These reports used English-speaking interviews with subtitles in the local languages. Recently, Friedmann et al. [8] reversed the design and used Russian-language interviews of the Hamilton Rating Scale for Depression and English subtitles. They demonstrated high concordance rates between the Russian and American raters.

The Montgomery-Asberg Depression Rating Scale (MADRS) is frequently used as a primary efficacy measure to assess patients with major depressive disorder (MDD) in multi-national clinical trials [9]. In their initial report, the authors’ asserted that the MADRS was culturally neutral although the instrument was only field tested in the UK and Sweden. The MADRS is used throughout the world, but the universality and cultural relevance of the 10 items have not been carefully assessed. However, the clinical relevance of the MADRS (or any other rating instrument) in clinical research or in practice is contingent upon the validity of the selected items for the culture being assessed. It is possible that some MADRS items may generate different interpretations and yield different scores in non-Western cultures.

The Japanese language and culture are distinctly different from countries in Western Europe or the USA. Historically, the existence of depression as an illness was suppressed in Japan, and suicide was often associated with honor rather than a consequence of social problems or depressive disease [10]. Clearly, these perceptions about depression are very different from the beliefs and attitudes commonly held in Western countries. In fact, until recently, there was still some reluctance to acknowledge major depressive disorder in Japan [10,11]. Long-held traditional beliefs about responsibility, honor, and productivity may influence the perception and scoring of some MADRS items like reported sadness, lassitude, inability to feel, pessimism, or even suicidal thoughts.

It was of some interest to examine the cross-cultural applicability of the MADRS in a Japanese population of depressed patients. Beyond the issue of inter-rater agreement and data comparability, cultural validity is an important issue whenever a rating instrument developed in one language is used in another. To evaluate the possibility that cultural differences may affect MADRS scoring, we conducted a rater training program comparing two MADRS videotaped interviews conducted in Japan and scored by Japanese and American raters. These MADRS interviews were administered in Japanese and were rated by US clinicians using the identical Japanese-language MADRS interviews with corresponding English subtitles.

We asked the following questions:

1. Can American clinicians using English subtitles achieve equivalent scores to Japanese clinicians on MADRS interviews conducted in Japanese?

2. Are there any individual items that reflect different cultural interpretations of the MADRS instrument?

## Methods

This study was conducted as part of a rater training and certification program developed for a clinical trial being conducted for patients with MDD in Japan. The rater certification program consisted of three components: didactic instruction about the MADRS, assessment of successful scoring accuracy on two MADRS demonstration video interviews, and assessment of research interviewing competency based upon satisfactory administration of a mock MADRS interview. The didactic training program for the MADRS was developed in English (SDT, Clintara, USA), translated into Japanese (AN, Keio University, Japan), and presented in Japanese at an investigator meeting held in Tokyo, Japan (February 2011) for these clinical trials.

The MADRS is a well-known 10-item symptomatic questionnaire developed over 30 years ago during the era of tricyclic antidepressants to assess clinical change during treatment [9]. The 10 MADRS items were chosen from the 65-item Comprehensive Psychopathological Rating Scale (CPRS) based upon their sensitivity to changes with treatment on four antidepressant drugs during a double-blind clinical trial of 54 English and 52 Swedish patients [9]. These 10 ‘best’ items included sadness (both reported and observed), psychic inner tension, reduced sleep, reduced appetite, concentration difficulties, lassitude, inability to feel, pessimism, and suicidal thoughts. Each of the 10 items has specific descriptors and scoring anchors to facilitate scoring and is scored from 1 to 6 with increasing evidence of severity. Total MADRS scores in the mid-20s generally reflect moderate depression, and scores in the mid-30s reflect moderate to severe depression [12,13].

In preparation for the rater-training program, five MADRS interviews were videotaped for evaluation purposes. The interviews were administered in Japanese (AN) with Japanese volunteer subjects using a structured interview guide (SIGMA) developed by Williams and Kobak [14] and subsequently subtitled in English. No informed consent was required because the subjects were volunteers acting the scripted role of depressed patients. The scripts were developed to reflect typical histories and clinical presentations of depressed patients in Japan. Each interview was transcribed into a Japanese script, translated into English by the interviewer himself (AN), and subsequently confirmed by an independent translator in the USA prior to subtitling in English. An effort was made to include in the subtitles the complete text of the original interview as it had been spoken in Japanese.

A panel of three Japanese raters from Keio University (Tokyo, Japan) and three US raters from the Massachusetts General Hospital (Boston, MA, USA) was convened before the investigator meeting to review each interview for comprehension, relevance to a Japanese population, and suitability for training and to establish an acceptable individual item score range and ‘gold standard’ total MADRS score. The consensus ‘gold’ score was used for the assessment of the proportion of inter-rater agreement (kappa statistic).

As planned, two MADRS interviews reflecting moderate to severe depression were selected as most suitable for the rater-training program and for the assessment of inter-rater agreement.

Subject 1 was a 35-year-old married woman whose preoccupation, sadness, and discouragement about her fertility problems affected work performance and social interactions due to her distractibility, sluggishness, shame, and loss of interest in friends and hobbies. The clinical presentation met DSM-IV criteria for major depressive disorder although it was clear that psychosocial stress was a confounding factor contributing to her mood disorder.

Subject 5 was a 40-year-old man who complained of constant sadness, fatigue, concentration difficulties, and excessive stress related to his inability to be more productive and advance in his job. This presentation of ‘fatigue depression’ is common in Japan and had clearly affected this subject’s interpersonal relations and well as his work performance.

The selected MADRS interviews (subjects 1 and 5) were shown to the Japanese raters at the investigator meeting. The Japanese clinician raters recognized these volunteer subjects as believable cases of depression consistent with the type of patients they had seen in their practice. Subsequently, a small group of experienced US raters (*n* = 23) observed and scored the same two videotaped interviews containing the corresponding English subtitles. The US raters did not speak or understand Japanese. Demographic data were collected from all raters including education, clinical experience, and rating experience with the MADRS instrument. Statistical analysis included Student’s *t* test comparison (with Bonferroni correction for multiple comparisons) and a kappa statistic to determine the proportion of inter-rater agreement based upon the gold standard consensus panel score [15,16].

## Results

### Demographics

Thirty-four Japanese raters and 23 US raters participated in this reliability study. The Japanese raters were all male physicians (mean age = 51.97 ± 8.08 years) with 22.7 ± 7.87 years of clinical experience (range 9 to 38 years) and 5.25 ± 7.87 years of MADRS rating experience (range 1 to 15 years). Eight of the Japanese raters had two or fewer years of MADRS rating experience. The US raters included male (*n* = 11) and female (*n* = 12) physicians, psychologists, and nurses (mean age = 45.54 ± 9.54 years) with 14.6 ± 5.81 years of clinical experience (range 5 to 42 years) and 6.15 ± 8.12 years of MADRS rating experience (range 4 to 15 years). None of the US raters spoke Japanese.

### Inter-rater agreement

Both the Japanese and US raters demonstrated high inter-rater agreement on the two MADRS interviews that were scored. The mean total MADRS score for the first interview (subject 1) was 33.31 ± 3.91 (SD) for the Japanese raters and 33.57 ± 3.04 for the US raters (*t* = 0.260; degrees of freedom (*df*) = 53; *p* = not significant (ns)). Similarly, the mean total MADRS score for the second interview (subject 5) was 29.76 ± 2.83 for the Japanese raters and 30.77 ± 2.64 for the US raters (*t* = 1.34; *df* = 54; *p* = ns). Analysis of the variance between the Japanese and US sets of MADRS ratings was also not significant for subject 1 (*F* = 1.68; *p* = ns) or subject 5 (*F* = 1.17; *p* = ns). Only four of the Japanese raters (12.9%) required rating remediation prior to meeting qualification requirements to rate in the clinical study. Among the US raters, there were no significant scoring differences related to education or between men and women raters.

**Figure 1:**
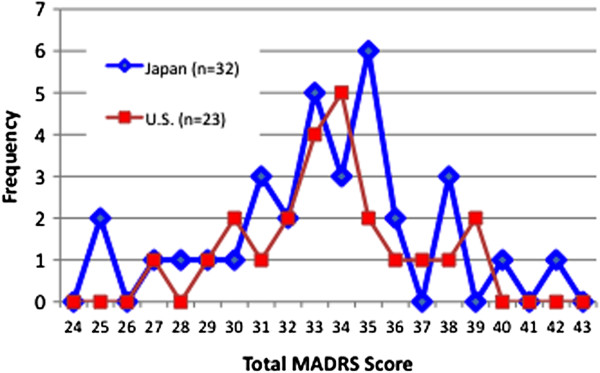
Total MADRS score distribution for subject 1.

**Figure 2:**
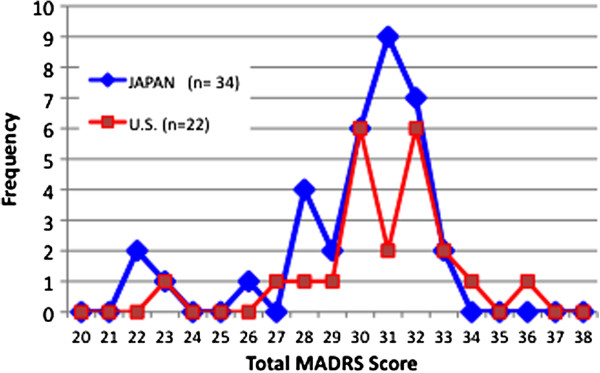
Total MADRS score distribution for subject 5.

Figures 1 and 2 reveal similar distributions of the total MADRS scores between the US and Japanese raters on both interviews. Of 54 raters, 45 (83%) were within one standard deviation of the median MADRS score for subject 1, and of 56 raters, 42 (76%) for subject 5. The US raters watching the Japanese-language MADRS interviews with English subtitles demonstrated high scoring concordance with the Japanese raters who watched the same interviews in their own language. The Kappa statistic measuring the proportion of agreement among all raters (as a categorical measure) was 0.92 for subject 1 and 0.86 for subject 5 using the accepted ‘standard’ scores previously derived by the consensus panel of three Japanese and three US raters.

### Individual MADRS item analyses

As shown in Tables 1 and 2, there were no significant scoring differences between the US and Japanese raters on the majority of individual MADRS items (using Bonferroni correction for multiple comparisons). However, the US raters scored lassitude (item 7) significantly higher (*p* = 0.013) and the inability to feel (item 8) significantly lower (*p* = 0.037) than Japanese raters on subject 1. There was also a non-significant trend on item 7 in subject 5 (*p* = 0.160).

**Table 1:** MADRS individual item analysis for subject 1

**MADRS item**^**a**^	**1**	**2**	**3**	**4**	**5**	**6**	**7**	**8**	**9**	**10**
Japanese raters										
Mean			3.28	3.72	4.25	2.56	3.16	3.84	3.53	1.69
STD	0.88	0.63	0.99	0.58	0.51	0.91	0.57	0.51	0.80	0.59
US raters										
Mean	3.83	4.00	3.39	3.39	4.04	2.43	3.83	3.39	3.43	1.83
STD	0.58	0.67	0.78	0.58	0.21	0.73	0.89	0.58	0.66	0.49
*T* value	−1.26	−1.58	−0.44	2.06	1.84	0.56	−3.40	3.04	0.47	−0.92
*P*^b^	ns	ns	ns	ns	ns	ns	*0.013*	*0.037*	ns	ns

^a^MADRS items: 1 (apparent sadness), 2 (reported sadness), 3 (inner tension), 4 (reduced sleep), 5 (reduced appetite), 6 (concentration difficulties), 7 (lassitude), 8 (inability to feel), 9 (pessimistic thoughts), 10 (suicidal thoughts). ^b^Bonferroni correction for multiple comparisons. *STD* standard, *ns* not significant.

**Table 2:** MADRS individual item analysis for subject 5

**MADRS item**^**a**^	**1**	**2**	**3**	**4**	**5**	**6**	**7**	**8**	**9**	**10**
Japanese raters										
Mean	3.82	3.97	3.41	2.41	0.12	3.91	3.29	4.09	3.24	1.50
STD	0.52	0.46	0.70	0.61	0.41	0.51	0.87	0.93	0.89	0.62
US raters										
Mean	3.73	4.05	3.59	2.68	0.00	4.14	3.82	4.32	3.32	1.14
STD	0.46	0.58	0.50	0.48	0.00	0.64	0.59	0.48	0.72	0.47
*T* value	0.71	−0.54	−1.04	−1.76	1.34	−1.45	−2.48	−1.07	−0.37	2.36
*P*^b^	ns	ns	ns	ns	ns	ns	*0.160*	ns	ns	ns

^a^MADRS items: 1 (apparent sadness), 2 (reported sadness), 3 (inner tension), 4 (reduced sleep), 5 (reduced appetite), 6 (concentration difficulties), 7 (lassitude), 8 (inability to feel), 9 (pessimistic thoughts), 10 (suicidal thoughts). ^b^Bonferroni correction for multiple comparisons. *STD* standard, *ns* not significant.

## Discussion

Contemporary clinical trials that pursue new drug development are often multi-national, involving multiple languages and diverse cultural groups. Researchers cannot presume that rating instruments developed and tested in Western countries can reliably assess illness severity and accurately measure serial change in patients from non-Western cultures. The issue of cross-cultural applicability is important in any clinical situation where rating instruments developed in one language are used in another. We examined the cross-cultural scoring agreement on one instrument (the MADRS) that was developed in two European countries as part of a clinical trial for patients with major depressive disorder. We conducted a comprehensive rater-training program that included two MADRS interviews administered in Japanese with Japanese subjects for certification of the Japanese raters. These same MADRS interviews were shown to the US raters with accompanying English subtitles to ascertain inter-rater scoring agreement and to explore possible cultural differences in interpretation and scoring.

The US and Japanese raters demonstrated high inter-rater agreement and no significant mean total MADRS scoring differences. Most raters scored within one standard deviation of the median MADRS score on both interviews. Clearly, the English subtitles did not adversely affect the high concordance observed between the US and Japanese scoring.

We analyzed the 20 individual items contained in the two MADRS interviews to explore possible interpretational differences between the US and Japanese raters. Although there were no significant scoring differences on 18 items (using a Bonferroni correction for multiple comparisons), two significant scoring differences between the rater groups were noted in subject 1. The US raters scored lassitude significantly higher (*p* = 0.013) and the inability to feel significantly lower (*p* = 0.037) than Japanese raters. Subject 5 revealed no significant item scoring differences. Subject 5 depicted a man presenting with overwork (fatigue) depression, a condition that is commonly seen in Japan but is not diagnosed or recognized by most American clinicians [10,11,17]. We found no significant scoring differences on depressed mood, anxiety, lassitude, or pessimism in this subject as scored by the US or Japanese raters. However, there was a non-significant trend (*p* = 0.160 with the Bonferroni correction) with US raters scoring higher than Japanese raters on the scoring of item 7 (lassitude) in subject 5.

It is reasonable to presume that Japanese raters are more attuned than US raters to the clinical relevance and nuance of a Japanese patient presenting with *expressed* lack of initiation (sluggishness), and *unexpressed* numbness (inability to feel). These scoring differences may reflect genuine cultural differences in the interpretation of the MADRS items. Alternatively, these scoring differences may reflect more about the cultural familiarity of the Japanese clinician with the clinical presentation of Japanese patients than to specific cultural beliefs about depressive mood, anxiety, lassitude, or the inability to feel.

The structured research interview format of the MADRS assesses the severity of depressive symptoms over the past 7 days and specifically dissociates diagnoses from the assessment [9,14]. The descriptors and scoring anchors relate to the specific symptoms and are meant to be generic guides. Further, the standardized methods used for investigator training seek to minimize any interpretational scoring differences by improving the precision of ratings. Therefore, we anticipated that cultural differences might be less likely to influence the MADRS scores. However, the findings in this small study suggest that there might be interpretational differences between US and Japanese raters on some MADRS items. The interpretation of lassitude or the inability to feel may be influenced by cultural views and may not be as neutral as the assessment of appetite or sleep. Of course, our findings must be interpreted with caution given its small size. We used only two MADRS interviews in this study that were conducted with two volunteer subjects (not patients with MDD) presenting with relatively similar depressive symptom severity (MADRS approximately 30 to 34). Clearly, our findings are limited by virtue of the small number of individual MADRS items assessed. It is conceivable that a larger sample of interviews might have revealed more cultural interpretational differences that were not uncovered in this small analysis. Further, our study was conducted in only one Asian country and may not be applicable to other Asian cultures.

However, given these limitations, our findings in this cross-cultural MADRS analysis between Japanese and US raters do provide some support for the usefulness of the MADRS to assess the overall severity of depressive symptoms in distinctly different cultural groups. The findings also highlight the importance of using a clinician rater who is familiar with the culture of the patient being assessed.

## Conclusion and future directions

Researchers and clinicians cannot automatically presume that rating instruments developed and tested in Western countries can accurately assess illness severity and measure serial change in patients from non-Western cultures. In this study, we found that the MADRS, an instrument originally developed and validated in the UK and Sweden, was reliable when scored by the Japanese raters in Japanese-language videotaped interviews and by the US raters observing subtitled English translations of these same interviews. Given the multi-national nature of global studies, it is reassuring to provide this degree of confirmation among diverse cultural groups. However, cultural familiarity between rater and patient is still important to achieve the most clinically relevant information for precise scoring. In fact, two MADRS items that may be susceptible to cultural interpretations (lassitude and the inability to feel) were scored significantly differently by the US and Japanese raters in one of these interviews. Our findings are based on only two interviews and suggest that more extensive research is warranted with real patients who are seen in real clinical settings.

## Abbreviations

MADRS: Montgomery-Asberg Depression Rating Scale; MDD: Major depressive disorder; PANSS: Positive and negative syndrome scale.

## Competing interests

SDT, MD has received honoraria or grants from the following: Acadia, Acumen, Alkermes Inc., Amgen, AstraZeneca, BioMarin, BrainCells Inc., CeNeRx, Cephalon, Clintara LLC, CTNI MGH, EnVivo Pharmaceuticals, Euthymics, Eli Lilly and Company, EnVivo Pharmaceuticals, Forest Research, Functional Neuromodulation Inc., GlaxoSmithKline, Johnson & Johnson PRD, INC Research, Methylation Sciences Inc., NeoSync, Neurophage, Novartis Pharmaceuticals, Nupathe, Parexel International, PRA International, Prana Biotechnology Ltd., ReViva, Roche Labs, Sophiris, Sunovion, Targacept, Theravance, Transcept, and Wyeth labs.

AN, MD, PhD received grants from Eli Lilly, Pfizer Inc., and Igaku-Shoin.

YS, MD, PhD received grants from Astra Zeneca, Pfizer, Sanofi-Aventis, GlaxoSmithKline, Eli Lilly, Abbott, Novartis, MSD, Asahi-kasei, Takeda, Daiichi-Sankyo, Japan Chemical Research, EPS, Taisho, Ajinomoto, Tasly, Parexel, and Icon.

## Authors’ contributions

All listed authors in this manuscript have contributed substantially to the design, execution, analysis, and writing of the manuscript. All authors read and approved the final manuscript.

## Authors’ information

SDT is the scientific director and founder of Clintara LLC (Boston MA) and on the consulting faculty at Massachusetts General Hopsital (Boston, MA, USA). AN is the director of the Translational Medical Center, National Center of Neurology and Psychiatry and on the faculty of the Department of Neuropsychiatry, Keio University School of Medicine, Tokyo, Japan. YS is the director of the Center for Clinical Research, Keio University School of Medicine, Tokyo, Japan.
